# Preferences for Psychological Therapy or Support Within an ARMS Psychological Therapies Trial: The Importance of Targeted Intervention for Unusual Sensory Experiences

**DOI:** 10.1111/eip.70035

**Published:** 2025-04-02

**Authors:** Jahnese Hamilton, Akansha Singh, Chris Gibbs, Nicola A. Barclay, Lauren Birkett, Charleen Boyle, Toby Brandon, Robert Dudley, Jochen Einbeck, Victoria Larry, Jennifer Simpson, Guy Dodgson, Charles Fernyhough

**Affiliations:** ^1^ Cumbria, Northumberland, Tyne and Wear NHS Foundation Trust Newcastle upon Tyne UK; ^2^ Queen's University Belfast Belfast UK; ^3^ Durham University Durham UK; ^4^ Northumbria University Newcastle upon Tyne UK; ^5^ University of York York UK; ^6^ Tees, Esk and Wear Valleys NHS Foundation Trust Darlington UK

**Keywords:** At Risk Mental State (ARMS), MUSE, Preferences for Psychological Help, Risk for Psychosis, Treatment Preferences

## Abstract

**Background:**

Individuals with an at risk mental state (ARMS) often experience hallucinatory‐type experiences, which we refer to as unusual sensory experiences (USE). However, it is not known whether individuals want to know more about USE or discuss these in therapy. Our preferences study asked whether individuals who are referred into a treatment trial for USE in ARMS consider attention to USE important.

**Methods:**

Ninety‐four service users of ARMS services within two UK National Health Service (NHS) mental health trusts completed the study‐specific, “Preferences for psychological therapy or support” questionnaire. Questions elicited preferences for target of therapeutic work and therapist approach. Analysis employs a repeated measures ANOVA with post hoc analysis of difference between preferences.

**Results:**

Treatment preferences which help understand *causes* of USE and how to *manage* USE were the group priority above talking therapy generally or a focus on low mood or anxiety. Provision of medication was the lowest priority in treatment preference though it was important to some. Overall, working with a therapist to make sense of experiences was more important than having space to talk, new ideas for coping, or working collaboratively on goals.

**Conclusions:**

Psychological intervention for individuals with at‐risk mental state needs to include acceptable and credible psychoeducation on causes of USE and how to manage these.

## 1 Introduction

At risk mental state (ARMS) is a term used to refer to people who commonly have a set of experiences and symptoms such as hallucinations or unusual sensory experiences (USE) that are indicative of psychosis‐like experiences but do not meet the threshold for a diagnosis of psychosis (Dudley et al. 2023; Yung et al. 2005). People experiencing subthreshold experiences can find these experiences distressing; higher levels of distress are associated with poorer outcomes (Nelson et al. 2022), and the person's associated distress and the impact of these experiences are key considerations for referral to ARMS services (Strelchuk et al. 2023).

ARMS services aim to offer full assessment along with needs‐based supportive psychological and psychosocial interventions in the community, to prevent possible progression to psychosis and other severe and enduring mental health problems (NHS England 2023; De Salazar Pablo et al. 2021). In England, ARMS services are structured within the wider healthcare system to provide a stepped care approach. Antipsychotic medication is not recommended, but medications for other co‐occurring medical diagnoses can be offered, such as for anxiety or depression (NHS England 2019, 2023; NICE 2013, 2014, 2021).

Whilst this detail of ARMS service structure and clinical guideline for intervention may suggest a robust system offering a helping hand to people who are in distress, patients and referrers alike report barriers to access and unmet needs, and report experiences of engaging in more generalised talking therapy services to be a poor match to their needs (Strelchuk et al. 2023). It is therefore important to consider this further from a patient perspective and ask individuals experiencing distress who come through the ARMS pathway what they consider important for meeting their needs.

Researchers have surveyed individuals accessing ARMS about some of these issues, a recent study of 54 individuals pre and post “risk for psychosis” assessment explored the use of this descriptor and found that it elicited positive and negative feelings associated with being assessed, along with some internal stigmatisation (Woodberry et al. 2021). This finding highlights the sensitivities around the language and concepts used in services with young people, particularly when stereotyped associations could trigger self‐stigma (Watson et al. 2007; Wood et al. 2017). However in another qualitative study, service users reported the ARMS descriptor and the accompanying explanation of their experiences helpful (Welsh and Tiffin 2012). This accompanying explanation may therefore be key to overcoming internal stigmatisation identified by Woodberry et al., as concurrent to becoming associated with the “stigmatised” group (risk of psychosis) individuals are supported with alternative non‐stigmatised appraisals of their experiences (Carter et al. 2017). Experiencing USE is a frequent reason for referral to ARMS services (Strelchuk et al. 2023) and is a symptom that elicits greater public stigmatisation in association with mental health challenges (Phalen et al. 2019), but just how important this focus is to individuals with ARMS requires further investigation.

In a feasibility trial (Hamilton et al. 2023) (results to be reported separately), we invited individuals from ARMS who were experiencing USE to take part in a feasibility trial of a novel intervention, which aims to give helpful explanations as to how USE occurs: The Managing Unusual Sensory Experiences (MUSE) intervention is a psychoeducational toolkit that can be used by therapists to present information on a screen; using images, videos, animations, audio, and written information. The toolkit provides research‐informed explanations of how the mind works, along with reflections from people with lived experience. It uses current psychological models to try to explain how hallucinations could occur, with a rationale that under strain, normal cognitive processes can break down and lead to hallucinations. Within the package is a selection of CBT‐informed formulation routes, with attention to different subtypes of USE presentation (inner speech, hypervigilance, memory, and visual) to support recognition and reflection of different mechanisms of USE. Further CBT‐informed tasks are accessible for individual adaptation to encourage the use of alternative coping strategies and to embed learning. Therapists select the pages appropriate to the individual's needs to review and discuss together in session, and for actions to explore between sessions. MUSE was developed in conjunction with extensive multidisciplinary research investigations into voice hearing and other experiences through the Hearing the Voice Programme (URL: hearingthevoice.org), and with the inclusion of input from individuals with lived experience (see Dudley et al. 2022, for further details on MUSE modules content). However, when inviting people into the trial we wanted to check their views on the importance of giving attention to explanations of USE; understanding these preferences is the aim of this paper.

People's thoughts and feelings towards treatment in mental health are important for their decision about engaging: Good collaboration with individuals that recognises preferences and enables a shared and informed decision for treatment can improve therapeutic relationships and lower resistance to help (Ferrari et al. 2024; Pérez‐Arechaederra et al. 2024; Simmons et al. 2021). Research has shown that alignment or misalignment of treatment with preferences can influence outcomes: This was demonstrated in a study involving individuals with sub‐threshold depression, where a positive relationship was identified between meeting treatment preference and subsequent outcomes (Cooper et al. 2018). In another larger national survey, the reverse relationship was also revealed with participants reporting less favourable outcomes where preferences for mental health treatment were not met (Williams et al. 2016). It is therefore imperative, particularly in the development of new treatments, to find out the preferences of people with ARMS: The provision of psychoeducational materials such as information leaflets, as well as CBT based intervention has shown some popularity (Welsh and Tiffin 2014) but the content of psychoeducational interventions is not always clearly defined or well tested in the literature (Herrera et al. 2023). CBT intervention may be better understood in terms of model and technique at least in the treatment of psychosis (Morrison and Barratt 2009), but even within CBT for psychosis there is a movement towards looking at the efficacy of adapting this to more symptom‐specific targeted intervention to enhance treatment effects (Lincoln and Peters 2019). Further to this, comprehensive reviews of the evidence for CBT in patients with ARMS highlight the need for improved research trials (Bosnjak Kuharic et al. 2019; Fusar‐Poli et al. 2019). As we develop, test and refine symptom‐specific psychological based interventions for individuals at risk of psychosis or with early onset (Smailes et al. 2015), such as the MUSE intervention (Dodgson et al. 2021a; Dudley et al. 2024; Hamilton et al. 2023), we need to ask patient's perspectives on the importance, or not, of paying attention to specific aspects of their experience, such as USE, and the distress that may be related to these presentation‐specific symptoms.

This study therefore asked individuals with USE who were accepted into ARMS services about their preferences for treatment. All participants who completed this questionnaire had also consented to take part in a randomised controlled feasibility trial of the MUSE intervention versus usual care (MUSE ARMS: Trial registration number ISRCTN58558617) (Hamilton et al. 2023). MUSE is currently undergoing investigations within trial settings with different clinical population groups to explore its usability in NHS settings and assess its potential treatment effects (Dodgson et al. 2021a, Dodgson et al. 2021b; Dudley et al. 2024; Hamilton et al. 2023). The outcome paper for the MUSE ARMS trial will be reported separately.

## 2 Materials and Methods

### 2.1 Public and Patient Involvement

This research was developed in collaboration with the study‐specific lived experience advisory panel (LEAP), who gave advice on the project development and design. The panel is made up of people with either lived experience of mental health difficulties and service use, and/or of caring for children/young adults with these experiences. The group (*n* = 7) had the opportunity to discuss the idea of asking for preferences from participants in MUSE ARMS, and they considered this important. They spent 90 min reviewing the wording of the preferences questionnaire, and changes were made to both question wording and the response options from this discussion. Further to this, two members of the panel trialed completion of the questionnaires with a researcher and fed back that the questionnaire felt appropriate in its final version. The results of the questionnaire and its analysis were fed back to the panel. One of the group is an author on this paper; other members are acknowledged personally or as a group as per their preferences. All other study‐specific participant materials, including participant information sheets and consent forms, were also co‐developed with the panel. In accordance with involvement good practise guidelines to support equality of inclusion, LEAP members were offered financial remuneration and expenses for their time (NIHR 2022).

### 2.2 Participants

Participants were patients accepted into ARMS services, aged 14–35 years, with hallucinations/unusual sensory experiences scoring at least three on the Perceptual Abnormalities Subscale (global rating item or frequency and duration item) of the Comprehensive Assessment of ARMS (CAARMS‐PA) (Yung et al. 2005), clinically stable, and with hallucinations to be considered a key target problem. Exclusion criteria were intellectual disability or severe cognitive dysfunction where it affected ability to engage with research materials, and/or lacking capacity to give informed consent. One‐hundred and thirty three people accessing ARMS services were approached over a 10‐month period from 11 ARMS teams within two secondary care NHS Trusts in North Cumbria and the North East of England. Ninety‐four participants consented and completed the measures.

### 2.3 Measures

A study‐specific questionnaire was developed in collaboration with the LEAP to collect data on participant preferences for psychological therapy or support (see Hamilton et al. 2023, Supporting Information, for the full questionnaire). The preferences questionnaire asks about: (1) Preference for number of sessions; answers are constrained to 1–3, 4–8, 9–16, 17–30, or do not know. (2) Preference for the treatment, to include talking therapy or medication, to focus on anxiety, low mood, understanding USE, managing USE, and/or reducing distress relating to USE; answers are selected from a choice of three responses, either: not important, somewhat important, or very important. (3) Preference relating to the way the therapist/clinical care team works, including giving space to talk and feel heard, working with the therapist to help make sense of experiences, being involved in setting own goals, and being given new ideas of how to cope with experiences; answers are selected from a choice of three responses, either: not important, somewhat important, or very important.

Sociodemographic details were collected using the CSRI Mental Health (Beecham and Knapp 2001), questions 1 to 3.5, as amended for the trial (Hamilton et al. 2023).

Clinical assessments assessed general functioning using the Social and Occupational Functional Assessment Scale (SOFAS) (Goldman et al. 1992). The SOFAS is a standardised assessment single‐item scale to assess social and occupational functioning; it is assessed by a clinician/clinical researcher using information from the interview. The period of assessment for this trial is the last two weeks. Scoring is from 0 to 100; higher scores represent better functioning.

USE were rated using the Comprehensive Assessment of At‐Risk Mental States—Perceptual Abnormalities subscale (CAARMS‐PA) clinical assessment (Yung et al. 2005), assessed by a clinician/clinical researcher using information from interview and medical records. This gives a global rating score of 0–6, which represents: 0 = never, absent; 1 = questionable; 2 = mild; 3 = moderate; 4 = moderately severe; 5 = severe; 6 = psychotic and severe. A frequency and duration score of 0–6 represents: 0 = absent; 1 = less than once a month; 2 = once a month to twice a week—less than 1 h per occasion; 3 = once a month to twice a week—more than 1 h per occasion or 3 to 6 times a week—less than 1 h per occasion; 4 = three to six times a week—more than an hour per occasion or daily—less than an hour per occasion; 5 = daily—more than an hour per occasion, or several times a day; 6 = continuous. The pattern of symptoms in relation to substance use is established, and a level of distress in relation to symptoms is scored between 0 and 100, 100 being the greatest. The SOFAS and CAARMS‐PA scoring by clinical researchers underwent inter‐rater reliability checks with a Clinical Psychologist specialising in ARMS assessments.

### 2.4 Procedure

NHS research ethical permissions were obtained from North East—Newcastle & North Tyneside 1 Research Ethics Committee (REC) (Reference: 23/NE/0032). All patients who were accepted into the ARMS services and who met eligibility criteria were invited to participate by a member of their clinical team. Those who expressed interest were contacted by the research team and given participant information, including the REC approved information sheets to read and discuss with family at least 3 days prior to giving informed consent. Participants gave informed consent to participate in the study, or assent with parental consent as applicable, prior to completing the study measures (for copies of consent/assent forms see Hamilton et al. 2023, supplement 5, 6 and 7). Research meetings took place at a location preferable to the participant, which could be their home, health centre, college or school.

## 3 Statistical Analysis

To explore hierarchical preferences in the preferences questionnaire for treatment and for therapy engagement, responses are scored as not important = 1, somewhat important = 2, very important = 3, to produce the overall mean scores for each question response. Analysis of the data is conducted using IBM SPSS Statistics (Version 27). A repeated measures ANOVA is conducted to test for significant differences between preference responses. Post hoc pairwise comparisons with Bonferroni correction is used to determine the strength of differences in preferences. Descriptive statistics for the number of sessions and preference for MUSE or TAU are reported in terms of frequency and percentages.

Descriptive statistics for sociodemographic characteristics are also reported using frequency and percentages. SOFAS scores are reported using range, mean ± standard deviation. CAARMS‐PA data for the global, frequency, and distress scores is reported using range, mean ± standard deviation. Relationship to substance use is reported using frequency and percentages.

## 4 Results

### 4.1 Participant Characteristics

**FIGURE 1:**
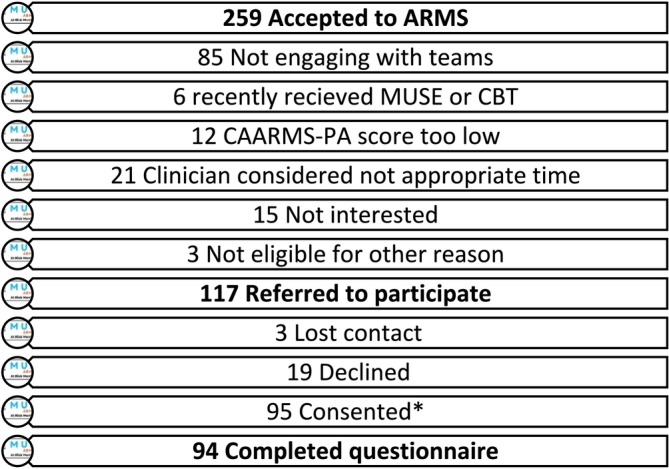
Accepted into ARMS services and referred to participate with attrition. *One participant met for consent but then disengaged.

There were 259 referrals accepted by the ARMS services over the recruitment period between April 2023 and February 2024. From this, 133 individuals were referred to the research team for potential inclusion. Reasons for non‐referral were varied, often relating to poor engagement or other pressing concerns prioritised by the clinical team. Only a small number could not be included because of a lack of sufficient USE (see Figure 1). From those referred, 94 participants who met the eligibility criteria provided informed consent and completed the preferences questionnaire. Gender figures (57.4% female, 40.4% male, 2.1% other) show a slightly higher number of females to males participating but were within the service norms, which ranged from 42% to 59% female acceptance rate across the services recruited from. Ethnicity is predominantly White‐British (91.5%), which reflects the regions recruited from; service audit data was examined for ethnic diversity and show a similar picture of average 93% White‐British across the services recruited from. Attrition from referrals to consents is also shown in Figure 1. Participants' sociodemographic characteristics are presented in Table 1.

**TABLE 1:** Sociodemographic characteristics.

Characteristics	94 Participants
Gender, *F* (%), *M* (%), other (%)	54 (57.4%), 38 (40.4%), 2 (2.1%)
Age (range, mean ± SD)	14–35, 19.5 ± 5.2
Ethnicity (%)	
Asian/Asian British	3 (3.2%)
Black/African/Black British	1 (1.1%)
White—any other white background	3 (3.2%)
White‐British	86 (91.5%)
Other ethnic background	1 (1.1%)
Education and employment	
In education/student	45 (47.9%)
In education and working	5 (5.3%)
Employed	15 (16%)
Not in education or employment (NEET)	29 (30.9%)
Living situation	
Living with parents	67 (71.3%)
Living alone (with or without children)	10 (10.6%)
Living with a partner or spouse	10 (10.6%)
Living with other relatives	1 (1.1%)
Living with others	6 (6.4%)
Estranged from family	10 (10.6%)
Housing type	
Owner occupied flat or house	32 (34.0%)
Privately rented flat or house	24 (25.5%)
Rented from local authority/housing association	33 (35.1%)
Homeless but not roofless	1 (1.1%)
Community overnight facility, 24 h staffed	1 (1.1%)
Other	3 (3.2%)

### 4.2 Clinical Characteristics

General functioning assessed with the SOFAS clinical assessment ranged from 31 to 90 (mean 58.26, SD = 14.52) for the sample, which indicates marked difficulties in social and occupational functioning across the majority of the sample. This is a slightly higher but generally comparative SOFAS mean score to those reported in other ARMS studies (Polari et al. 2021; Rekhi et al. 2019).

USE assessed with the CAARMS‐PA subscale showed global rating scores between 2 and 6 (mean 4.5, SD = 0.73), frequency and duration ranged from 2 to 6 (mean 4.34, SD = 1.05), which suggests that on average USE was moderately severe and occurred several times a week (Yung et al. 2005). Most participants reported USE to have no relationship to substance use (*n =* 80, 85.1%), and some reported USE to occur in relation to substance use and at other times as well (*n =* 14, 14.9%). CAARMS‐PA level of distress in relation to symptoms ranged from 0 to 100 (mean 70.14, SD = 20.42) with one missing data item due to a participant being unable to answer the question. Only two participants said they had no distress related to the USE. These CAARMS‐PA mean scores for global rating, frequency, and associated distress relating to USE are high, and higher than those reported in other studies sampling from a wider whole ARMS population (Rekhi et al. 2019; Wilson et al. 2020). Possible reasons for this are considered in the discussion.

### 4.3 Preferences for Psychological Therapy or Support

#### 4.3.1 Preference for Number of Sessions

Preference for the number of sessions was largely “don't know” (*n =* 56, 59.6%), followed by 17–30 sessions (*n =* 21, 22.3%), with fewer participants selecting 9–16 sessions (*n =* 5, 5.3%), 4–8 sessions (*n =* 4, 4.3%), and 1–3 sessions (*n =* 8, 8.5%).

#### 4.3.2 Preference for Treatment

Participant responses for preferences for treatment show mixed responses, which indicate value in the approach of ARMS services to make needs‐based assessment and provision (NHS England 2023). A visual inspection of the data shows highest preference being understanding causes of USE and learning how to manage USE (see Table 2 for results).

**TABLE 2:** Responses to preference for treatment,”how important is it that your treatment” questions, with order of columns matched to order of questions and overall Mean (*M*) and standard error (SE) scores.

	Includes being given medication (%)	Includes a talking therapy (%)	Addresses any feelings of anxiety (%)	Addresses any feeling of low mood (%)	Helps you understand the causes of any unusual sensory experiences, such as hearing a voice (%)	Helps you learn to manage any unusual sensory experience (%)	Helps you feel less distressed about any unusual sensory experiences (%)
Not important	26 (27.7%)	15 (16.0%)	6 (6.4%)	7 (7.4%)	2 (2.1%)	1 (1.1%)	3 (3.2%)
Somewhat important	43 (45.7%)	27 (28.7%)	29 (30.9%)	21 (22.3%)	8 (8.5%)	10 (10.6%)	14 (14.9%)
Very important	25 (26.6%)	52 (55.3%)	59 (62.8%)	66 (70.2%)	**84 (89.4%)**	**83 (88.3%)**	77 (81.9%)
Mean score (SE)^a^	1.99 (0.08)	2.39 (0.08)	2.56 (0.06)	2.63 (0.06)	2.87 (0.041)	2.87 (0.038)	2.79 (0.05)

^a^ Where scoring is: 1 = Not important; 2 = Somewhat important; 3 = Very important.

A repeated measures ANOVA and pairwise comparisons with Bonferonni adjustment examined these preference responses further. This identified significant differences between the responses to the questions, F(6,88) = 21.899, *p* < 0.001. The items in the pairwise comparisons significantly favoured were the treatment, “Helps you understand the causes of any unusual sensory experiences, such as hearing a voice” (mean 2.87, SE 0.041) and, “Helps you learn to manage any unusual sensory experiences” (mean 2.87, SE 0.038) above all other items. These were significantly higher than the preference for medication (*p* < 0.001), talking therapy (*p* < 0.001), addressing any feeling of anxiety (*p* < 0.001), and addressing any feeling of low mood (*p* = 0.009, *p* = 0.012), though not significantly higher than for the related question, “Helps you feel less distressed about any unusual sensory experiences” (*p* = 1.00, *p* = 0.94) (see Table 3). Preference that the treatment includes medication was the least important item (mean 1.99, SE 0.08), and this was significantly lower than all other preferences related items (*p* < 0.001). The final item “Helps you feel less distressed about any unusual sensory experiences?” (mean 2.79, SE 0.05) was significantly more important than overall preference for talking therapy (*p* < 0.001) and addressing feelings of anxiety (*p* = 0.04) but was not significantly more important than preference for addressing feelings of low mood (*p* = 0.81). Post hoc repeated measures analysis for the two age groups 14–17 and 18–35 years showed these findings applied for both age groups.

**TABLE 3:** Pairwise comparisons significance data for preference for treatment.

	Includes medication	Includes talking therapy	Addresses anxiety	Addresses low mood	Helps understand the causes of USE	Helps manage USE	Helps feel less distressed about USE
Includes medication		*M* = −0.404, SE = 0.098, *p* = 0.002	*M* = −0.574, SE = 0.085, *p* < 0.001	*M* = −0.638, SE = 0.083, *p* < 0.001	*M* = −0.883, SE = 0.083, *p* < 0.001	*M* = −0.883, SE = 0.080, *p* < 0.001	*M* = −0.798, SE = 0.086, *p* < 0.001
Includes talking therapy	*M* = 0.404, SE = 0.098, *p* = 0.002		*M* = −0.170, SE = 0.088, *p* = 1.000	*M* = −0.234, SE = 0.100, *p* = 0.441	*M* = −0.479, SE = 0.078, *p* < 0.001	*M* = −0.479, SE = 0.087, *p* < 0.001	*M* = −0.394, SE = 0.090, *p* < 0.001
Addresses anxiety	*M* = 0.574, SE = 0.085, *p* = < 0.001	*M* = 0.170, SE = 0.088, *p* = 1.00		*M* = −0.064, SE = 0.066, *p* = < 1.00	*M* = −0.309, SE = 0.068, *p* = < 0.001	*M* = −0.309, SE = 0.068, *p* = < 0.001	*M* = −223, SE = 0.070, *p* = < 0.038
Addresses low mood	*M* = 0.638, SE = 0.083, *p* = < 0.001	*M* = 0.234, SE = 0.100, *p* = 0.441	*M* = 0.064, SE = 0.066, *p* = 1.00		*M* = −0.245, SE = 0.067, *p* = 0.009	*M* = −0.245, SE = 0.069, *p* = 0.012	*M* = −0.160, SE = 0.076, *p* = 0.812
Helps understand the causes of USE	*M* = 0.883, SE = 0.083, *p* = < 0.001	*M* = 0.479, SE = 0.078, *p* = < 0.001	*M* = 0.309, SE = 0.068, *p* = < 0.001	*M* = 0.245, SE = 0.067, *p* = 0.009		*M* = 0.000, SE = 0.040, *p* = 1.00	*M* = 0.085, SE = 0.054, *p* = 1.00
Helps manage USE	*M* = 0.883, SE = 0.080, *p* = < 0.001	*M* = 0.479, SE = 0.087, *p* = < 0.001	*M* = 0.309, SE = 0.068, *p* = < 0.001	*M* = 0.245, SE = 0.069, *p* = 0.012	*M* = 0.000, SE = 0.040, *p* = 1.00		*M* = 0.085, SE = 0.042, *p* = 0.943
Helps feel less distressed about USE	*M* = 0.798, SE = 0.086, *p* = < 0.001	*M* = 0.394, SE = 0.090, *p* = < 0.001	*M* = 223, SE = 0.070, *p* = 0.038	*M* = 0.160, SE = 0.076, *p* = 0.812	*M* = −0.085, SE = 0.054, *p* = 1.00	*M* = −0.085, SE = 0.042, *p* = 0.943	

There were no significant differences between the items for preferences that the treatment includes talking therapy (mean 2.39, SE 0.08), focuses on anxiety (mean 2.56, SE 0.06), or focuses on low mood (mean 2.63, SE 0.06) for the whole group (see Table 3). Post hoc repeated measures analyses for the two age groups 14–17 and 18–35 years showed the younger age group to prefer treatment to address anxiety and low mood more than simply including talking therapy (*p* = 0.02).

#### 4.3.3 Preference for the Approach of the Therapist/Clinical Care Team

Participant responses were mixed, with value rated across the items. A visual inspection shows most participants considered working with their therapist to help make sense of their experiences as very important (see Table 4 for results).

**TABLE 4:** Responses for preference for the way the therapist/clinical care team works together questions, with the order of columns matched to the order of questions and overall Mean (*M*) and standard error (SE) scores.

	I am given space to talk and feel heard (%)	I work with my therapist to help me make sense of my experiences (%)	I am involved in setting my own goals (%)	I am given new ideas of how to cope with my experiences (%)
Missing	0 (0%)	0 (0%)	0 (0%)	1 (1.1%)
Not important	1 (1.1%)	0 (0%)	10 (10.6%)	2 (2.1%)
Somewhat important	25 (26.6%)	16 (17.0%)	47 (50.0%)	22 (23.4%)
Very important	68 (72.3%)	**78 (83%)**	37 (39.4%)	69 (73.4%)
Mean score (SE)^a^	2.71 (0.05)	2.83 (0.04)	2.29 (0.07)	2.70 (0.05)

^a^ Where scoring is: 1 = Not important; 2 = Somewhat important; 3 = Very important.

A repeated measures ANOVA and pairwise comparisons with Bonferonni adjustment also examined preference for the approach of the therapist/clinical care team. Repeated measures ANOVA analysis identified significant differences between the responses to the questions, F(3,91) = 19.11, *p* < 0.001. Pairwise comparisons showed participants' favoured working with a therapist to make sense of experiences (mean 2.83, SE 0.04) significantly more than the other three items of being given space to feel heard (mean 2.71, SE 0.05; *p* = 0.04), being given new ideas of how to cope with experiences (mean 2.70, SE 0.05; *p* = 0.04), and being involved in setting own goals (mean 2.29, SE 0.07; *p* < 0.001). Being involved in setting own goals was the least important preference and significantly lower than the other items (*p* < 0.001). There was not a significant difference between preference for space to be heard and new ideas of how to cope (*p* = 1.00) (see Table 5). Post hoc repeated measures analysis for the two age groups 14–17 and 18–35 years showed these findings applied for both age groups, with exception of the difference between “working with a therapist to make sense of experiences” and “being given space to feel heard”, for the younger age group being non‐significant.

**TABLE 5:** Pairwise comparisons significance data for preferences for the way the therapist/clinical care team works.

	Given space to talk and feel heard	Work together to make sense of experiences	Involved in setting my own goals	Given new ideas of how to cope with my experiences
Given space to talk and feel heard		*M* = −0.117, SE = 0.042, *p* = 0.042	*M* = 0.426, SE = 0.073, *p* = < 0.001	*M* = 0.011, SE = 0.060, *p* = 1.00
Work together to make sense of experiences	*M* = 0.117, SE = 0.042, *p* = 0.042		*M* = 0.543, SE = 0.072, *p* = < 0.001	*M* = 0.128, SE = 0.046, *p* = 0.040
Involved in setting my own goals	*M* = −0.426, SE = 0.073, *p* = < 0.001	*M* = −0.543, SE = 0.072, *p* = < 0.001		*M* = −0.415, SE = 0.076, *p* = < 0.001
Given new ideas of how to cope with my experiences	*M* = −0.011, SE = 0.060, *p* = 1.00	*M* = −0.128, SE = 0.046, *p* = 0.040	*M* = 0.415, SE = 0.076, *p* = < 0.001	

## 5 Discussion

This study aimed to find out if individuals accessing ARMS services who agreed to come into a treatment trial exploring a psychoeducational CBT‐based intervention had specific preferences for treatments that focus on USE. Participants were indeed help‐seeking and had marked levels of distress in relation to USE, as expressed on the CAARMS‐PA distress item (mean = 70.14, SD = 20.42), which was higher than in other studies in the field (Rekhi et al. 2019; Wilson et al. 2020). Potentially, this was because we recruited a sub‐sample of people in ARMS services who identified as being affected by USE, and therefore the findings from this study should be interpreted within this context. Participants also reported moderate to severe impairment of social and occupational functioning, as reflected by their SOFAS scores (mean 58.26, SD = 14.52). This is comparative to that reported in other ARMS studies (Polari et al. 2021; Rekhi et al. 2019), and offers insight into the challenges faced by this subclinical population.

The results in our study show a range of preferences, which highlights diversity in individual needs, but there is also a clear and significant overall priority for understanding the causes of USE and for help on how practically to manage USE. Preference for the way the therapist/clinical care team works also prioritised help to make sense of experiences over other more general support, coping, or goal‐focused approaches. This evidence highlights the importance of explicit attention to USE, supporting the rationale for clinical services to have specialist knowledge of USE and how to manage them (Yung et al. 2021). This was echoed by our lived experience panel. The findings therefore counter suggestions for psychotic‐type experiences to be de‐emphasised (Moritz et al. 2019), or for specialist services to be de‐prioritised (Ajnakina et al. 2019; Murray et al. 2021). The younger age group's need to not only work together to make sense of experiences but also be given space to talk and feel heard further emphasises the importance of taking time to provide safe spaces for individuals in this age bracket (14–18) whose mental health is at risk of deteriorating. This feedback supports mental health care approaches which aim to break down barriers to access for potentially help‐seeking adolescents (e.g., Boonstra et al. 2024; Rickwood et al. 2019).

Whilst CBT trials appear to have made the most progress in demonstrating benefit for the ARMS population, there is still concern regarding the limits to the efficacy of CBT and a need for more rigorous trials that examine symptom‐targeted and stepped care approaches (Bosnjak Kuharic et al. 2019; Hutton and Taylor 2014; Mei et al. 2021; Schmidt et al. 2015; Stafford et al. 2013). More explicit psychoeducation has been called for (Herrera et al. 2023), with the provision of precise, respectful and hopeful information on underlying mechanisms driving hallucinatory type experiences (Freeman 2024). The present study adds a sub‐set of ARMS service‐user voices to the discussion, evidencing a subjective need for focused interventions that help individuals understand and manage USE.

## 6 Strengths and Limitations

The focus on peoples' opinions of their needs regarding intervention, and the check on preferences prior to participating in a trial of a novel intervention is a clear strength of this study. Preferences indicated here support the rationale for a symptom‐targeted psychological intervention and align to the focus of ARMS services on USE, which is often a core referral reason for ARMS referral, assessment and treatment (NHS England 2023; Strelchuk et al. 2023; Yung et al. 2005). However, not all individuals accessing ARMS services may be experiencing USE or share the desire to focus on this. Whilst the current study demonstrates a group priority to focus on USE in a specific sample of people where USE was the target problem, the range of individual responses also indicates a variety of personal preferences which need to be considered on an individual basis.

The focus of the preferences questionnaire in this study was limited in scope, and further research using qualitative or mixed methods approaches could be used to produce more nuanced and in‐depth findings. The quantitative approach we used allowed for statistical analysis of the significance of results, but within this interpretation, it is important to recognise the individual differences across the sample too. The questionnaire used to elicit preferences was relatively brief to reduce task demands on participants who were completing additional trial measures. In further relation to the scope of this study, whilst these findings counter suggestions for psychotic‐type experiences to be deemphasised (Moritz et al. 2019) the sample included in this study was a sub‐sample selected for involvement in an intervention trial for USE. As such, broader samples may find alternative results.

In consideration of other discussions in the wider literature on priorities for intervention, we did not ask participants about interventions relating to substance use (de Meiros et al. 2024; Murray et al. 2021). Substance use was reported by 14.9% of participants in this study, indicating a relationship with substances for part of the sample, but a large majority of participants had experienced USE without any perceived relationship to substances.

Sociodemographic information reveals mixed backgrounds in terms of gender (male, female, and two other gender), education (current status and level of completion), employment or unemployment, family dependence or independence, and housing demographics. Ethnic diversity is low but is representative of the region and local ARMS services from which participants were recruited (North Cumbria and the North East of England), which is less ethnically diverse than some areas of the UK (ONS. 2022). However, this is a limitation to the generalisability of the current study in respect of the wider ARMS population voice, and we recognise the need for more research with individuals from ethnically diverse backgrounds to address concerns of individuals meeting ARMS criteria in individuals from racially and ethnically minoritised backgrounds (Byrne et al. 2019) where experiences of racism and migration bring specific stressors (Lazaridou et al. 2023), and where barriers to service access may exist (Huff et al. 2024; Kirkbride et al. 2012; Nerhus et al. 2015). Further research with more diverse samples would provide important information to understand potentially different priorities. Challenges to service access are widely documented; in an umbrella review of challenges young people face in accessing mental health care, Huff et al. (2024) highlight barriers relating to trust, support, accessibility, and finance, which are particularly prominent for individuals from racially diverse communities, refugees and immigrants. We recognise that providers and researchers need to take responsibility for measuring equity of access and where applicable, addressing inequalities in local contexts (Huff et al. 2024). The sample in the current study is representative of the local population served, but the findings are limited by a lack of strong representation from people from racially and ethnically minoritised backgrounds. Accessing individuals with racial or ethnic diversity who meet the ARMS criteria could be done by using structural approach to recruitment strategies to enhance racial and ethnic diversity within samples.

A further characteristic of potential interest within the current study was the levels of family estrangement in participants (10.6%), which connects with other research that has identified concerning levels of instability in the living situation of young help‐seeking individuals (Filia et al. 2021). A limitation of the present study is that this was not studied further; no questions were asked about preferences for family intervention (in relation to education about USE or other needs). However, the suggestion of wider social issues adds justification to recommendations for taking a needs‐based approach to support individuals accessing ARMS services (De Salazar Pablo et al. 2021). These topics e.g. estrangement, education/employment instability, housing instability and housing environment could be explored further in future research. In working with individuals in ARMS services, it is important to continue to consider the breadth of presentation and needs, including and beyond USE, and to continue to develop and robustly evaluate improvements in therapeutic interventions.

## 7 Conclusions

Psychological interventions upstream (within ARMS services) that acknowledge USE and provide accessible explanations on the causes of USE and how to manage them e.g. MUSE (Dodgson et al. 2021b; Hamilton et al. 2023) may have the potential to (a) improve service users understanding of these experiences and (b) meet service users priority need for support, as evidenced by the current study.

## Data Availability

The data that support the findings of this study are openly available in Durham University Collections at https://collections.durham.ac.uk/files/r2np193924v, reference number doi:10.15128/r2np193924v.
